# Changes in Gut Microbiota Correlates with Response to Treatment with Probiotics in Patients with Atopic Dermatitis. A Post Hoc Analysis of a Clinical Trial

**DOI:** 10.3390/microorganisms9040854

**Published:** 2021-04-15

**Authors:** Eric Climent, Juan Francisco Martinez-Blanch, Laura Llobregat, Beatriz Ruzafa-Costas, Miguel Ángel Carrión-Gutiérrez, Ana Ramírez-Boscá, David Prieto-Merino, Salvador Genovés, Francisco M. Codoñer, Daniel Ramón, Empar Chenoll, Vicente Navarro-López

**Affiliations:** 1 Biopolis S.L.-ADM, Catedrático Agustín Escardino Benlloch 9 Edif. 2, 46980 Paterna, Spain; eric.climent@adm.com (E.C.); Juan.MartinezBlanch@adm.com (J.F.M.-B.); laura.llobregat@adm.com (L.L.); Salvador.genoves@adm.com (S.G.); Francisco.codoner@adm.com (F.M.C.); Daniel.ramonvidal@adm.com (D.R.); 2 Department of Clinical Medicine, Universidad Católica San Antonio de Murcia (UCAM), 30107 Murcia, Spain; beatriz.ruzafa@bioithas.com (B.R.-C.); aaramirezbosca@gmail.com (A.R.-B.); vnavarro@ucam.edu (V.N.-L.); 3 Especialida des Farmacéuticas Centrum, Calle Sagitario 14, 03006 Alicante, Spain; miguelcarrion65@gmail.com; 4 Department of Dermatology, Hospital Universitario Vinalopó, 03293 Elche, Spain; 5 Applied Statistical Methods in Medical Research Group, Universidad Católica San Antonio de Murcia (UCAM), 30107 Murcia, Spain; dprieto@ucam.edu; 6 Faculty of Epidemiology and Population Health, London School of Hygiene and Tropical Medicine, London 400706, UK; 7 Clinical Microbiology and Infectious Disease Unit, Hospital Universitario Vinalopó, 03293 Elche, Spain

**Keywords:** atopic dermatitis, gut-skin axis, microbiome, probiotics, *Faecalibacterium*, *Bifidobacterium*

## Abstract

Atopic dermatitis (AD) is a chronic recurrent inflammatory skin disease with a high impact on the comfort of those who are affected and long-term treated with corticosteroids with limited efficacy and a high prevalence of relapses. Because of the limited effectiveness of these treatments, new strategies for recovery from AD lesions are continually being explored. In this article, we describe the gut microbiome changes achieved in a recently published clinical trial with the probiotic formulation *Bifidobacterium animalis* subsp. *lactis* CECT 8145, *Bifidobacterium longum* CECT 7347, and *Lacticaseibacillus casei* CECT 9104 (formerly *Lactobacillus casei* CECT 9104), showing a significant improvement in SCORAD (scoring atopic dermatitis) index in children (4–17 years) with AD (Clinicaltrials.gov identifier: NCT02585986). The present gut microbiome post hoc study showed no significant changes in diversity (Shannon and Simpson indexes) after probiotic consumption. In the probiotic group, genera *Bacteroides, Ruminococcus*, and *Bifidobacterium* significantly increased their levels while *Faecalibacterium* decreased, compared to the placebo group. *Faecalibacterium* showed the highest presence and significant positive correlation with AD severity (SCORAD index), whereas *Abyssivirga, Bifidobacterium,* and *Lactococcus* were inversely correlated. The results suggest that the consumption of the probiotic formulation here assayed modulates the gut microbiome with significant changes in genera *Bacteroides* and *Faecalibacterium*. In turn, the improvement in SCORAD correlates with a decrease in *Faecalibacterium* and an increase in *Bifidobacterium*, among others.

## 1. Introduction

Atopic dermatitis (AD) is a chronic recurrent inflammatory skin disease with a high impact on the comfort of those who are affected since it produces intense pruritus, inflammation, and skin-barrier disruption, which provokes difficulty falling asleep and embarrassing or angry feeling about their appearance. Its highest prevalence appears in children, accounting for up to 20% infant population around the world [1,2]. This disease is increasing its prevalence, characterized by early onset (before 5 years old), being frequent in infants younger than 1 year [3]. Although there is a genetic risk background, mostly derived from filaggrin gene (FLG) mutations [4], impairing of skin-barrier function, and increased risk of AD, it is well-known the crucial role of immunity. In AD, an imbalance between Th2 cells and Th1 occurs, which might aggravate its pathogenesis, increasing immunoglobulin E (IgE) and activating interleukins [5]. In addition, interleukin IL-17 has been reported to reduce expression of FLG and involucrin [6], whereas IL-31 can induce pruritus in patients via natriuretic peptide release [7].

Culture-dependent microbiology analysis in AD skin lesions has identified an increased *Staphylococcus aureus* colonization in comparison with non-lesioned areas affecting severity, being toxin-producing *St. aureus* strains more prevalent [8]. Compromised barrier integrity, altered sphingolipid metabolism, and antimicrobial peptide expression are hypothesized to facilitate colonization with *St. aureus* [9,10] and specific fungi [11]. Massive sequencing platforms have led to deeply analyzed the role of microbiota in AD pathogenesis. As in the case of the culture-dependent approach, several studies have analyzed the relationship between skin microbiota and AD. Based on next-generation sequencing approaches, it has been found a low diversity in AD patients, together with *St. aureus* increase and also reduction in genera *Acinetobacter*, *Corynebacterium, Prevotella*, *Propionibacterium,* and *Streptococcus* [12,13].

However, new findings suggest that the digestive microbiome may affect host health not only locally but beyond the gut. In a state of balance (eubiosis), the digestive microbiome actively participates in the metabolic and physiological processes of the host, providing key molecules and enzymatic processes within the ecosystem. When this microbiome is out or unbalanced (dysbiosis), either as a cause or as a consequence of a disease process, the microorganisms present, or those that are no longer present, can modify the patterns of this ecosystem. It has been reported that there is a close bidirectional interface between the intestine and the liver (gut-liver axis) through the biliary tract, portal vein, and systemic circulation [14]. Moreover, if we look further, there is now robust evidence showing a communication route between the digestive microbiome and the central nervous system, via the enteric nervous system, with neurotransmitters, neuro-regulators, and hormones playing different roles [15]. Within this activity, the integrity of the intestinal barrier is clearly a target of action of the digestive microbiome, providing protection in a state of eubiosis and affecting its integrity in dysbiosis by leading to local inflammation and bacterial translocation [14].

In recent years, the relationship between the gut microbiome and skin health is being discussed, pointing to a gut microbiome-skin axis. It is hypothesized that the gut microbiome can in early life affect immune system maturation through cross-talk between the microbiome and the host, which could impact AD development [16,17] via inflammation caused by specific bacteria [18]. Short-chain fatty acids (SCFAs) have been shown to act in the AD gut due to their anti-inflammatory and immunomodulatory effects [19] and their role in maintaining the aforementioned barrier integrity [20], which has been directly related to the origin of AD [21].

Topical corticosteroids have been the keystone of pharmacological treatments for slight to moderate AD [22], although long-term data for these medications lack for pediatric patients [23]. Systemic drugs such as immunosuppressants are more effective than their topical counterparts but have substantially severe side effects and risks of rebound after treatment discontinuation [24].

Because of the side effects of these treatments and the frequent relapses, new strategies for recovery from AD lesions are continually being explored. On the one hand, strategies based on the topical use of compounds with bactericidal capacities, such as ozone, have been assayed [25]. On the other hand, and due to the relationship between the gut microbiome and AD, new ways of improvement are being analyzed from the modification of the present dysbiosis. In this regard, we recently published a study in which the use of a blend of probiotics with anti-inflammatory properties in children (4–17 years) with AD achieved a significant improvement in the SCORAD (scoring atopic dermatitis) index [26]. Based on that clinical trial, a post hoc study has been carried out to evaluate the impact of the use of probiotics on the gut microbiota, with the aim of identifying markers of severity and improvement. The present study describes the results found.

## 2. Materials and Methods

### 2.1. Study Population

Samples came from ATO/PRO1 AD clinical trial (Clinicaltrials.gov Identifier NCT02585986) run in Hospital General Universitario de Alicante (Alicante, Spain) [26]. Briefly, children aged from 4 to 17 years with moderate AD [27] were recruited between March and June 2016. All patients met the Hanifini and Raijka criteria for AD, and the severity of AD was measured using the SCORAD index. Exclusion criteria were as follows: use of systemic corticosteroids, methotrexate, cyclosporine, or anti-tumor necrosis factor drugs in the previous 3 months, antibiotics in the previous 2 weeks, or concomitant diagnosis of intolerance to gluten and/or lactose or signs of bacterial infection, among others [26]. A peripheral blood sample was collected from all participants and analyzed for routine biochemical laboratory values and for interleukin levels. Finally, stool samples were obtained at the beginning and at the end of the study for massive parallel sequencing.

### 2.2. Protocol

The study design was a randomized, double-blind placebo-controlled trial. Patients were enrolled in the study (randomization ratio 1:1) and received daily a pill with probiotic formulation (10^9^ colony-forming units (CFUs) of a mixture of the probiotic strains *Bifidobacterium animalis* subsp. *lactis* CECT 8145, *Bifidobacterium longum* CECT 7347, and *Lacticaseibacillus casei* CECT 9104 (formerly *Lactobacillus casei* CECT 9104 [28]) with maltodextrin as a carrier, in a 1:1:1 ratio), or placebo (only maltodextrin) for 12 weeks. The SCORAD index score was measured at the time of inclusion and then every four weeks until the end of the 12-week intervention period. Further protocol is described in Navarro-López et al. [24].

### 2.3. Assignment and Intervention

All patients received standard treatment during the 12-week study period according to the clinical guidelines for the management of AD [29]. Participants in the probiotic group received daily a pill containing 10^9^ total cfu of the mixture of the three probiotic strains [26]. Investigators and assessors collecting outcome data were blinded for the assigned intervention during the study.

### 2.4. Microbiome Determinations

Fecal samples were collected at baseline and after 12 weeks of treatment and stored at −80 °C until analysis. DNA from stool samples was isolated following Yuan et al. [30], adding bead beating and enzymatic lysis steps prior to extraction to avoid bias in DNA purification toward misrepresentation of Gram-positive bacteria, with the aid of Magna Pure Compact System (Roche Diagnostics, Barcelona, Spain). The composition of the gut microbiome was evaluated by massive parallel sequencing of the hypervariable region V3-V4 of the bacterial 16s rRNA gene. Amplification was run with eubacterial primers [31] and sequence achieved by MiSeq Illumina Platform (Illumina inc, San Diego, CA, USA), following the Illumina recommendations. The resulting sequences were split per patient. PEAR program version 0.9.1 was applied to overlap R1 and R2 reads, with an overlap of 50 nts and a minimum quality of Q20 [32], providing a single FASTQ file for each of the samples. Quality control of the sequences was performed: (i) quality filtering (minimum threshold of Q20) using fastx tool kit version 0.013, (ii) primer trimming and length selection (reads over 300 nts) was performed with cutadapt version 1.4.1 [33]. FASTQ files were converted to FASTA files, and UCHIME program version 7.0.1001 was used to remove chimeras. Clean FASTA files were BLAST against NCBI 16s rRNA database using blastn version 2.2.29+. The resulting XML files were processed using a python script developed by ADM Biopolis (Valencia, Spain) to annotate each sequence at different phylogenetic levels. Violin plots were produced using ggpubr library in R (https://CRAN.R-project.org/package=ggpubr, access date 18 January 2021).

### 2.5. Statistical Analysis

Statistical analyses were produced using R version 3.3.0. Normality of the microbiome data was discarded using a Shapiro test, and the differences between groups at microbiome level were tested with a paired Wilcoxon Mann–Whitney test. All the diversity indexes were obtained with the vegan package, their normality distribution was confirmed with a Saphiro test, and the differences between groups were tested with an ANOVA test. Changes in the microbiome associated with SCORAD values were measured with DESeq2 library, adjusting a negative binomial model with SCORAD as a numeric covariate.

## 3. Results

### 3.1. Definition of Core Microbiome

A total of 50 patients previously diagnosed with AD by clinical, laboratory, and/or histological findings were randomized in the trial [24]. We were able to obtain and analyze fecal samples at baseline and after 12 weeks from 43 patients (21 into the placebo group and 22 in the probiotic group).

Firstly, the intestinal bacterial composition in AD patients was analyzed by massive parallel sequencing, inspecting an average of over 68,000 raw sequences per sample (raw and clean number of sequences, mean length, total megabases sequenced, and mean quality per sample can be found at Supplementary Table S1). Rarefaction curves were produced with vegan package in R in order to measure the bacterial community coverage. All curves had similar levels of saturation, implying that all microbiomes have been equally covered and can be compared between them. Table S2 shows microbiome characterization per each sample and taxonomic level.

Enterotypes composition was studied [34] (Table 1). At baseline, enterotype 1 (predominance of *Bacteroides*) was described by 51.16% of patients (22/43), enterotype 3 (predominance of *Ruminococcus*) 44.18% (19/43), while enterotype 2 (predominance of *Prevotella*) accounted for only 4.60% (2/43).

### 3.2. Microbiome Evolution

The microbiome was studied in AD patients after 12 weeks of either placebo or probiotic treatment and changes from basal samples analyzed. At baseline, no differences in bacterial composition were found between patients in the placebo group and those in the probiotic group (Figure S1).

Table 1 shows contingency analysis for enterotype evolution. Around 40% of enterotype 1 AD patients changed to other enterotypes, regardless of the treatment (Pearson’s chi-squared test *p*-value = 0.754). The two patients with enterotype 2 at baseline changed to enterotype 3. Of the 11 patients classified as enterotype 3 at baseline in the probiotic group, 5 (45%) changed to enterotype 1. While of the eight patients with enterotype 3 at baseline in the control group, only 2 (25%) changed to enterotype 1. In addition, changes from enterotype 1 to 3 occurred in both groups, but no differences between groups were statistically significant (Pearson’s chi-squared test *p*-value = 0.3437).

**Figure 1:**
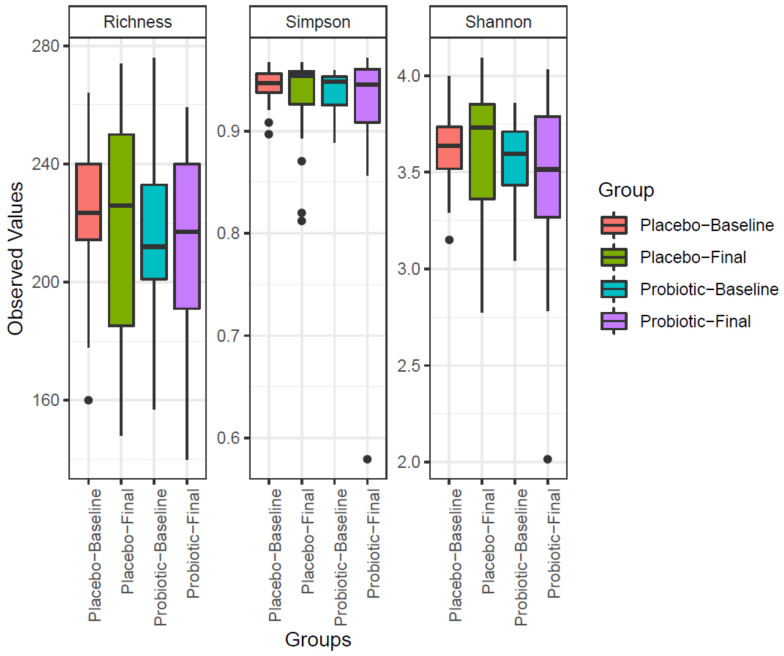
Diversity values obtained in placebo and probiotic groups.

Three different indexes were obtained using vegan package of R: Richness and diversity Shannon and Simpson (Figure 1). Once the normality distribution was confirmed (Shapiro test), the ANOVA test was applied to evaluate the changes between placebo and probiotic groups, resulting in not significant.

**Figure 2:**
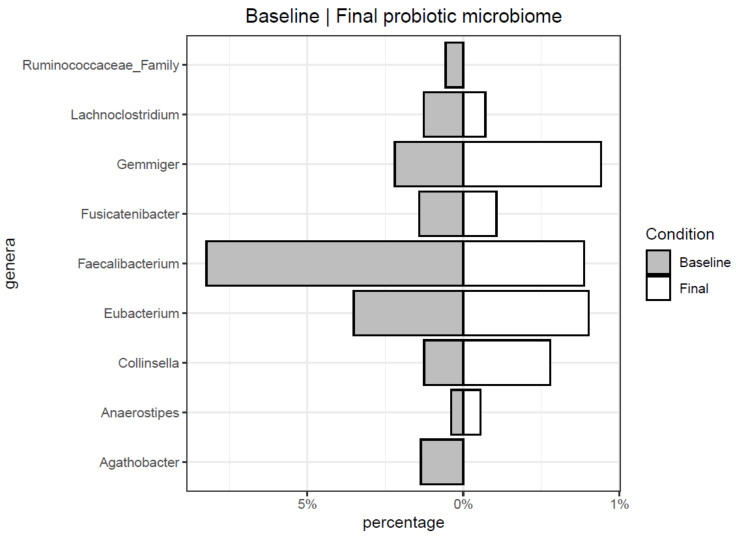
Significant genera before and after probiotic treatment.

**Figure 3:**
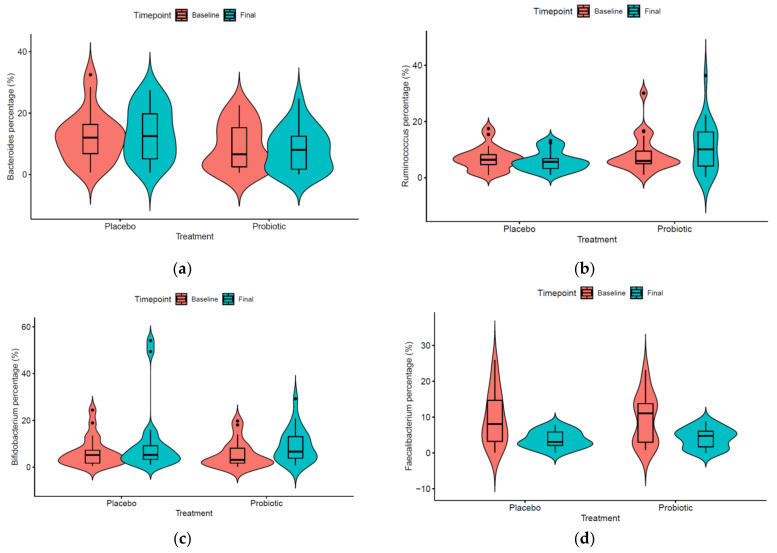
Violin plots showing significant genera comparing the placebo and probiotic groups’ evolution: (**a**) genus *Bacteroides*; (**b**) genus *Ruminococcus*; (**c**) genus *Bifidobacterium*; (**d**) genus *Faecalibacterium*.

Each of the principal genera was studied individually in order to check if the treatment had some significant effect. First of all, the normality presumption of the data was discarded using a Shapiro-Wilk normality test. All comparisons were produced with non-parametric Kruskal–Wallis test and Wilcoxon rank-sum test. Figure 2 shows the taxa that significantly differed in children consuming a probiotic product. Genera *Agathobacter*, *Faecalibacterium*, *Fusicanibacter*, and *Lachnoclostriudium*, and one unidentified representative of the *Ruminococcaceae* family decreased after 12 weeks of probiotic consumption. On the other hand, members of genera *Anaerostipes*, *Collinsella*, *Eubacterium*, and *Gemmiger* increased their levels. When the effect of probiotic consumption compared to placebo at the final time is analyzed (Figure 3), specific taxa evolution is revealed. Genera *Bacteroides*, *Bifidobacterium*, and *Ruminococcus* significantly increased their levels while *Faecalibacterium* decreased Conversely, in the case of *Bifidobacterium* species, presumptive *B. longum*, B. *bifidum*, and *B. pseudocatenulatum* OTUs were the most abundant, but none of them increased significantly in the probiotic group compare to placebo (Kruskal and Wilcoxon test). In the case of *Faecalibacterium*, *F. prausnitzii* was identified at the species level.

### 3.3. Correlation between SCORAD Index and Microbiome Profile

**Figure 4:**
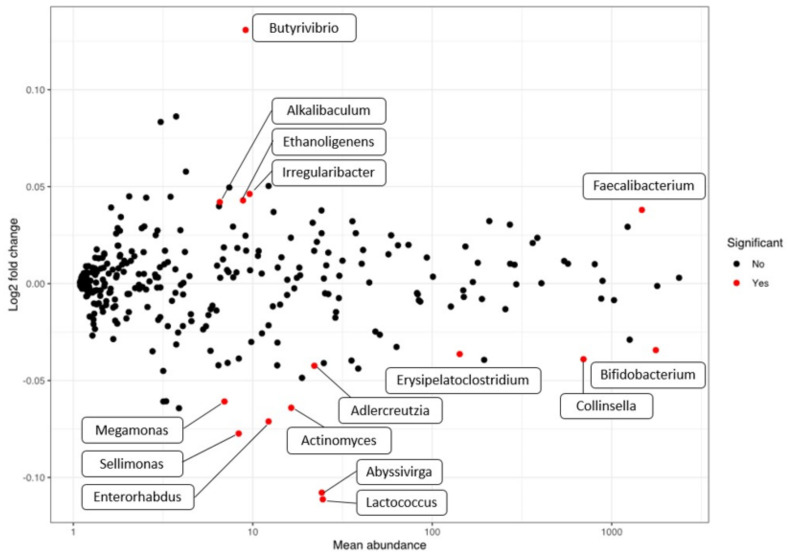
Correlation between SCORAD index and genera.

With the aim of detecting specific biomarkers among the 16S rRNA microbiome that could correlate with the SCORAD index, independently of the treatment received and time of sampling, DESeq2 was applied (Figure 4 and Table S3). SCORAD index was used as a continuous numerical covariable in the DESeq2 model, and the resulting log2FoldChange implies the change in each genus when SCORAD increases 1 point, considering both baseline and final data. When we analyzed taxa that directly correlate with SCORAD index (and consequently with AD severity), phylum Actinobacteria was inversely correlated (log2Foldchange = −0.04) and consequently inversely correlated with AD severity score. At family level, none was directly correlated whereas *Actinomycetaceae* (log2Foldchange = −0.006, *p*-value < 0.01), *Eggerthellaceae* (log2Foldchange = −0.04), *Selenomonadaceae* (log2Foldchange = −0.06) and *Bifidobacteriaceae* (log2Foldchange = −0.04) were inversely correlated with AD severity, being *Bifidobacteriaceae* the taxon with highest presence (basemean = 1766.16 sequences). At genus level (Figure 4), *Butyrivibrio* showed the major log2Foldchange (log2Foldchange = 0.13), and *Faecalibacterium* showed the highest presence and significant positive correlation with AD severity-microbiome (log2Foldchange = 0.04, basemean 1472.37 sequences). On the contrary, genera *Abyssivirga* and *Lactococcus* provided the highest negative correlation (inversely correlated with AD severity), being log2Foldchange = −0.11 in both cases. *Bifidobacterium* rendered a log2foldchange = −0.03 and the highest basemean among the significant populations (basemean = 1761.01 sequences).

## 4. Discussion

The gut is the most important source of postnatal microbial stimulation of the immune system, and atopic children may have a different gut microbiome compared with their non-atopic peers. Although it has been claimed a gap of information in school-age children as microbiome studies are concentrated in early infants and adults [35], differences have been found between cases of eczema and healthy controls [36] as well as between countries with a high and low incidence of atopic diseases [21,37]. In a clinical study aimed to evaluate the effect of a probiotic consortium (*B. animalis* subsp. *lactis* CECT 8145, *B. longum* CECT 7347, and *L. casei* CECT 9104) in AD, we demonstrated its effectiveness in improving SCORAD and diminishing the use of topical steroids in 4–17 years old children [26]. In a very recent published meta-analysis of the effectiveness of probiotic strains for the treatment of pediatric AD, this consortium was described as the most effective one [38]. Since gut dysbiosis has been observed in patients with AD [21,39], we wanted to evaluate whether the positive effect of this probiotic consortium could be due to some extent to a modulation of the microbiome toward a closer to healthy profile. The present work delves into the study of the evolution of the gut microbiome resulting from the consumption of the probiotic.

Alpha-diversity evolution was analyzed at the species level. In previous studies with AD children, diversity was recorded lower than a healthy population [39]. In our study, neither Shannon nor Simpson indexes did significantly change after probiotic consumption.

It is openly discussed whether the functional ability of probiotics is mainly related to their capacity to resist the digestive tract, arrive in the gut biologically active, and colonize [14]. In our study, when microbiome evolution is analyzed, lactobacilli and presumptive *B. animalis* are not detected increased in the probiotic group. The improvement in SCORAD obtained in the probiotic group [26], despite not increasing the abundance of probiotics’ OTUs in the probiotic group samples, indicates that other mechanisms beyond a simple increase in the bacterial groups consumed might be acting and that, even probiotics were partially protected in gelatin capsules avoiding oral interferences, the digestive tract is impacting probiotics’ colonization.

In the probiotic group, its consumption is accompanied by a decrease in *Faecalibacterium* concentrations. This genus has been previously related to the AD gut microbiome in children [39]. Song and coworkers [21] reported a model in which AD is associated with an imbalance in *F. prausnitzii* non-butyrate producer strains and defined by the release of nutrients in damaged intestinal mucosa and subsequently gut permeability maintenance and aberrant Th2-type immune responses in the skin [21]. In the case of *Bacteroides*, the consumption of the probiotic increased the basal levels found. In the intestine, *Bacteroides* spp. are able to ferment complex sugars into a number of byproducts, including SCFAs, such as acetate, butyrate, formate, and propionate [40]. Lower levels of this genus could imply fewer production of these compounds and consequently a more inflammatory status. In a previous study, Rios-Covian et al. demonstrated that certain exopolysaccharides (EPS)-producing bifidobacteria (*B. animalis* subsp. *lactis* and *B. longum* strains) were able to stimulate the growth of *Bacteroides* by feeding their EPS suggesting a role for these biopolymers in bacteria-bacteria cross-talk within the intestine [41]. In our study, both species are included in the probiotic consortium, and even though we did not detect a significant increase in their levels, they have the capacity to produce EPS (material not intended for publication: Silva et al., Biopolis S.L.-ADM, Paterna, Spain, screening of EPS-producing strains, 2014), so this might be one of the mechanisms for impacting gut microbiota dysbiosis, not only based on a direct increase of their levels but also working as microbiome modulators.

The results of our clinical study indicated a strong positive effect in reducing the severity of AD on the basis of the SCORAD index [26]. When we analyze the correlation between the SCORAD index and specific populations, neither phylum nor family were positively correlated with the SCORAD index. Phylum Actinobacteria and families *Actinomycetaceae*, *Bifidobacteriaceae*, *Eggerthellaceae*, and *Selenomonadaceae* were slightly inversely correlated with SCORAD and subsequently positively related with AD improvement. At the genus level, even though *Butyrivibrio* showed the highest levels of change, its abundance proved to be very low, so it may not have an effect on SCORAD. The genus *Faecalibacterium*, however, also showed a positive correlation with SCORAD and a large presence, which points to it as a marker of severity in AD. Both genera are capable of producing butyrate using the pyruvate pathway [42] and showed as commensals in healthy gut studies [43,44]. Since they are capable of producing the anti-inflammatory molecule butyrate as a product of their metabolism, there may be acting a mechanism that selects non-producing strains. An alternative and less plausible explanation may lie in the potential effect of high butyrate concentrations on intestinal apoptosis and disruption of the intestinal barrier, as has been described by Huang and coworkers [45]. New studies will be needed to analyze the role of producing and non-butyrate-producing *Faecalibacterium* strains modulation in the production of SCFAs and further in the improvement of SCORAD in the AD population after probiotic consumption. The recently described genus *Abyssivirga* and also the genus *Lactococcus* provided the highest negative correlation with SCORAD, although their presence levels were rather low, which would, in principle, rule out a critical role as biomarkers. *Bifidobacterium* was the genus with the highest presence and with negative SCORAD correlation, linking with the increase in phylum Actinobacteria detected. This normal inhabitant of healthy infants and adults’ gut has widely described beneficial roles. Most of them are associated with the prevention and treatment of intestinal diseases and immunological disorders such as IBD or necrotizing enterocolitis and also in allergic symptoms by modulating the Th1/Th2 balance and IgE [46], among others, which links the increase in bifidobacteria with lower SCORAD severity index. Although the correlation values that have been seen are slight, they do sketch out a rough outline of what the marker bacteria might be in this AD gut microbiome and point to a modulating effect of the probiotic as a source of its functionality in children with AD. Finally, no modifications of the microbiota that could potentially be associated with health concerns were observed. In this sense, not only the effectiveness but also the safety of probiotic ingestion must be assured in studies since there is evidence that certain strains (either by probiotic consumption or by contamination of products) have caused health problems [14].

All these results indicate that the consumption of the probiotic blend here assayed modulates the gut microbiome with significant changes in genus *Bacteroides* and, in turn, lower SCORAD scores correlate with a decrease in *Faecalibacterium* and an increase in *Bifidobacterium*. However, there are still limitations to this work, the most notable being the reduced number of the study, to be increased in future work, and the translation of these results to children of lower ages and adults. In addition, the question of whether the changes in the gut microbiome that are detected are a consequence of the use of probiotics or purely due to the improvement of the disease is a matter of future studies.

## Figures and Tables

**Table 1:** Contingency enterotype analysis in both probiotic and placebo groups.

	Placebo				Probiotic			
Baseline\Final	1	2	3	%	1	2	3	%
1	7	1	4	57.14	6	1	3	45.46
2	0	0	1	4.76	0	0	1	4.54
3	2	0	6	38.10	5	1	5	50.00
%	42.86	4.76	52.38		50.00	9.09	40.91	

## Data Availability

Raw 16S sequences in fastq format can be found in the Sequence Read Archive (SRA) under the accession reference PRJNA699431.

## Supplementary Materials

The following are available online at https://www.mdpi.com/article/10.3390/microorganisms9040854/s1, Figure S1: PCA global AD study; Table S1: raw and clean number of sequences, mean length, total megabases sequenced, and mean quality per sample can be found at Supplementary; Table S2: microbiome characterization per each sample and taxonomic level; Table S3: DEseq2 study microbiome vs. SCORAD score.
