# Supplementary material for: Changes in Gut Microbiota Correlates with Response to Treatment with Probiotics in Patients with Atopic Dermatitis. A Post Hoc Analysis of a Clinical Trial
Source: Microorganisms. 2021 Apr 15;9(4):854. doi: 10.3390/microorganisms9040854 (PMC8071520; doi:10.3390/microorganisms9040854)
Supplement: Supplementary file 1 [file microorganisms-09-00854-s001.zip › Supplementary/Figure S1_PCA AD Global.pdf]

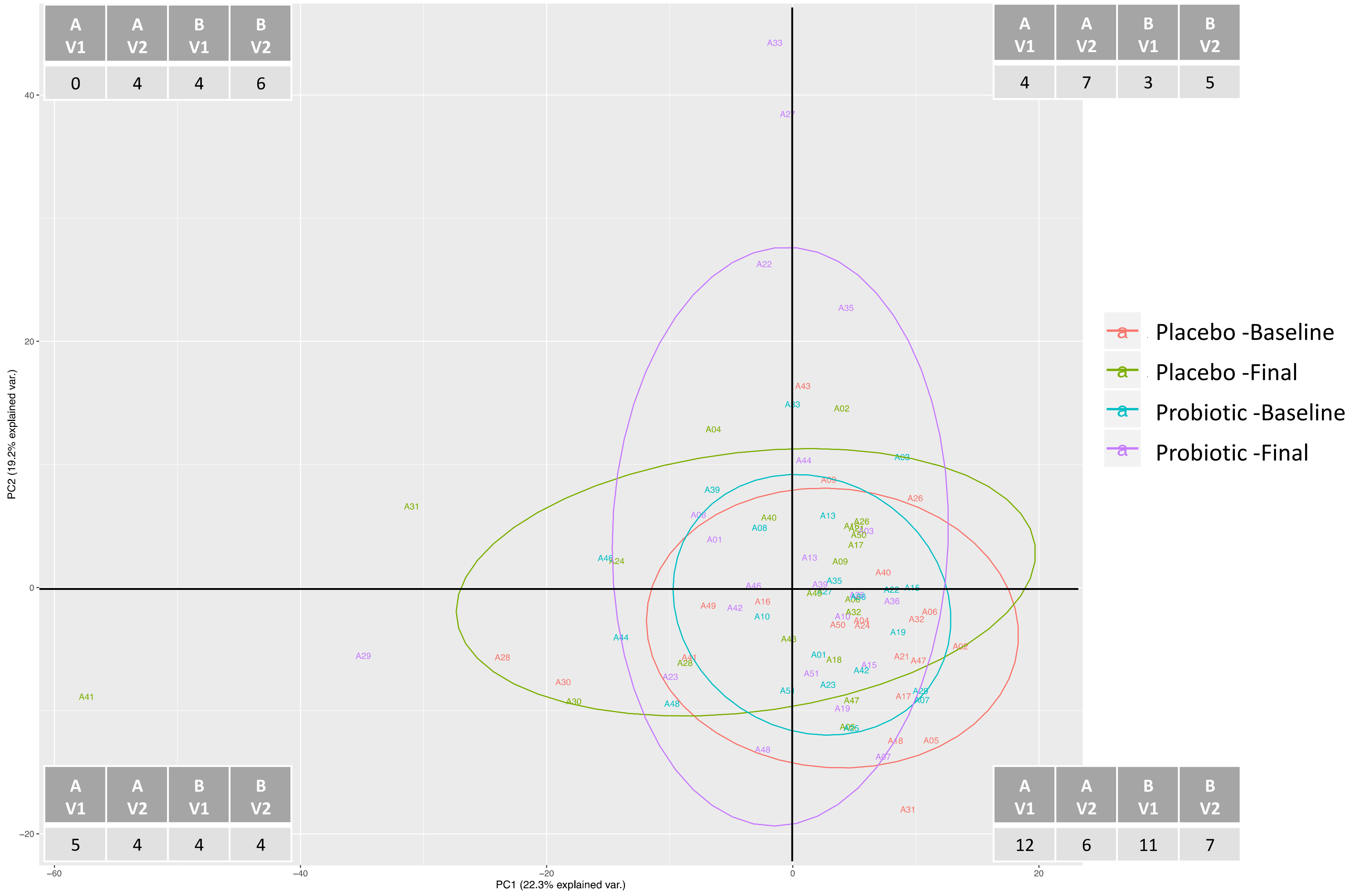

	PC1
**Akkermansia**	−0.900340965787125
**Bifidobacterium**	−0.0557043486993092
**Anaerobacterium**	−0.0467889211025036
**Ruminococcus**	0.14154761132399
**Faecalibacterium**	0.29253993945556

	PC2
**Bacteroides**	−0.478061763161603
**Faecalibacterium**	−0.140151659544938
**Akkermansia**	−0.134516595645746
**Ruminococcus**	0.127135824956158
**Collinsella**	0.17803730554487
**Bifidobacterium**	0.789722819744763

Figure S1. PCA global dermatitis study.
